# Combined effectiveness of prior and current season influenza vaccination in northern Spain: 2016/17 mid-season analysis

**DOI:** 10.2807/1560-7917.ES.2017.22.7.30465

**Published:** 2017-02-16

**Authors:** Jesús Castilla, Ana Navascués, Itziar Casado, Jorge Díaz-González, Alejandra Pérez-García, Leticia Fernandino, Iván Martínez-Baz, Aitziber Aguinaga, Francisco Pozo, Carmen Ezpeleta, Primary Health Care Sentinel Network, Network for Influenza Surveillance in Hospitals of Navarre

**Affiliations:** 1 Instituto de Salud Pública de Navarra, IdiSNA - Navarre Institute for Health Research, Pamplona, Spain; 2 CIBER Epidemiología y Salud Pública (CIBERESP), Spain; 3 Complejo Hospitalario de Navarra, IdiSNA - Navarre Institute for Health Research, Pamplona, Spain; 4 Centro Nacional de Microbiología (WHO National Influenza Centre - Madrid), Instituto de Salud Carlos III, Majadahonda, Spain; 5 The members of these networks are listed at the end of the article

**Keywords:** influenza, influenza vaccine, influenza-like illness, case control study, vaccine effectiveness, repeated vaccination

## Abstract

The 2016/17 mid-season vaccine effectiveness estimate against influenza A(H3N2) was 15% (95% confidence interval: −11 to 35) in Navarre. Comparing to individuals unvaccinated in the current and four prior seasons, effectiveness was 24% for current and 3–4 prior doses, 61% for current and 1–2 prior doses, 42% for only current vaccination, and 58% for 3–4 prior doses. This suggests moderate effectiveness for different combinations of vaccination in the current and prior seasons.

During the early 2016/17 influenza season, influenza A(H3N2) was the main circulating virus in Europe [1]. Although most of the A(H3N2) viruses characterised genetically matched the vaccine component, a high incidence of severe cases was detected [1,2]. We present the 2016/17 mid-season vaccine effectiveness (VE) estimates in preventing laboratory-confirmed influenza A(H3N2), relative to different combinations of current and prior seasonal influenza vaccinations.

## Setting and information sources

In the 2016/17 season the A(H3N2) component recommended for the influenza vaccine in the northern hemisphere was A/HongKong/4801/2014-like virus (group 3C.2a) [3], in the 2015/16 season A/Switzerland/9715293/2013-like (group 3C.3a) [4], and in seasons 2012/13 to 2014/15 it was A/Texas/50/2012-like or A/Victoria/361/2011-like (group 3C.1) [5]. 

The Influenza Surveillance System in Spain reported that as of 9 February 2017, 99% of the sentinel detections of influenza virus were A(H3N2), and sequence analysis of the HA1 fragment of the haemagglutinin gene found 74% of strains as A/Bolzano/7/2016 (group 3C.2a1) and 21% as A/HongKong/4801/2014, both of which matched the vaccine component [2].

A test-negative case–control study was conducted, based on epidemiological and virological surveillance of influenza in primary healthcare and hospitals in Navarre, northern Spain. The influenza vaccination campaign took place in October and November 2016. The trivalent inactivated non-adjuvanted vaccine was offered free of charge to a target group for vaccination, including people aged 60 years or over and those with major chronic conditions (body mass index ≥40 kg/m^2^, cancer, liver cirrhosis, dementia, diabetes mellitus, immunodeficiency, heart disease, renal disease, respiratory disease, rheumatic disease, and stroke).

Influenza vaccine status in seasons 2012/13 to 2016/17 was obtained from the online regional vaccination register [6]. These five seasons were considered because for all of them the A(H3N2) component included in the vaccine belonged to clade 3C [3-5]. 

Patients were considered to be protected from influenza 14 days after vaccine administration in the current season.

Influenza surveillance was based on automatic reporting of cases of influenza-like illness (ILI) from all primary healthcare physicians and hospitals [7]. A sentinel network of primary healthcare physicians was requested to take nasopharyngeal and pharyngeal swabs from their patients diagnosed with ILI, whose symptoms had begun less than five days previously. In hospitals, the protocol specified early detection and swabbing of all hospitalised patients with ILI. Samples were processed by reverse transcription-polymerase chain reaction assay.

## Statistical analysis

The study population included persons covered by the Navarre Health Service since 2012 (96% of the population). All patients who were swabbed between 1 December 2016 (beginning of continued detection of influenza virus) and 31 January 2017 were initially considered. Healthcare workers, persons living in nursing homes, children less than 9 years-old and patients hospitalised before ILI symptom onset were excluded. We compared seasonal vaccination status in patients for whom A(H3N2) influenza virus was detected (cases) and in those who were negative for influenza (controls). Crude and adjusted odds ratios (OR) with their 95% confidence intervals (CI) were calculated using logistic regression models. Adjusted models included sex, age group (9–24, 25–44, 45–64, 65–84 and ≥ 85 years), major chronic conditions, month of swabbing, and healthcare setting (primary healthcare and hospital). Six categories combining current vaccination status with vaccination in the four prior seasons and thus distinguishing between frequent and infrequent vaccinees were considered [8,9]: current-season vaccination and > 2 prior doses, current-season vaccination and 1–2 prior doses, current-season vaccination and no prior doses, no current-season vaccination and > 2 prior doses, no current-season vaccination and 1–2 prior doses, and no current-season vaccination and no prior doses (reference group). To compare VE among categories, the model was repeated using the category with current season vaccination and > 2 prior doses as the reference. VE was estimated as a percentage: (1–OR) × 100.

## Early estimation of influenza vaccine effectiveness

Of 1,243 ILI initial patients, one case of non-subtyped influenza A and two influenza B cases were not further considered. The remaining 1,240 ILI patients were included in the study and consisted of 783 (63%) hospitalised patients and 457 primary healthcare patients. A total of 591 (48%) were confirmed cases for influenza A(H3N2) and were compared with 649 controls negative for any influenza virus. 

Compared with test-negative controls, A(H3N2) influenza cases had a lower proportion of persons over 65 years-old (53% (315/591) in cases vs 62% (401/649) in controls; p = 0.003), with major chronic conditions (59% vs 71%; p < 0.001; Table 1) and who were treated in hospital (51% (300/591) vs 74% (483/649; p < 0.001)). Among the cases, 41% had received the 2016/17 seasonal vaccine, vs 50% of the controls (p = 0.001) (Table 1).

**Table 1:** Characteristics, according to the healthcare setting and test result, of patients with medically-attended influenza-like illness included in the test-negative case–control analysis, Navarre, Spain, 1 December 2016–31 January 2017 (n = 1,240 patients)

Characteristics	All patients				Hospitalised patients				Primary healthcare patients			
	Controls		Cases		Controls		Cases		Controls		Cases	
	N	%	N	%	N	%	N	%	N	%	N	%
**Age groups in years**												
9–24	37	6	56	9	14	3	1	0	23	14	55	19
25–44	76	12	99	17	22	5	7	2	54	33	92	32
45–64	135	21	121	20	80	17	33	11	55	33	88	30
65–84	269	41	197	33	236	49	143	48	33	20	54	19
≥ 85	132	20	118	20	131	27	116	39	1	1	2	1
**Sex**												
Male	331	51	274	46	269	56	151	50	62	37	123	42
Female	318	49	317	54	214	44	149	50	104	63	168	58
**Residence**												
Rural	237	37	213	36	213	44	154	51	24	15	59	20
Urban	412	63	378	64	270	56	146	49	142	86	232	80
**Major chronic conditions**												
No	189	29	242	41	78	16	43	14	111	67	199	68
Yes	460	71	349	59	405	84	257	86	55	33	92	32
**Month of swabbing**												
December	159	24	139	24	106	22	58	19	53	32	81	28
January	490	76	452	76	377	78	242	81	113	68	210	72
**Target group for vaccination^a^**												
No	124	19	182	31	36	7	11	4	88	53	171	59
Yes	525	81	409	69	447	93	289	96	78	47	120	41
**2016/17 season vaccine**												
No	327	50	351	59	205	42	113	38	122	73	238	82
Yes	322	50	240	41	278	58	187	62	44	27	53	18
**Total**	**649**	**100**	**591**	**100**	**483**	**100**	**300**	**100**	**166**	**100**	**291**	**100**

^a^ Target group for vaccination includes people ≥ 60 years old and people with major chronic conditions (body mass index ≥40 kg/m^2^, cancer, liver cirrhosis, dementia, diabetes mellitus, immunodeficiency, heart disease, renal disease, respiratory disease, rheumatic disease and stroke).

The overall adjusted estimate of influenza VE was 15% (95%CI: –11 to 35). The estimates were similar in the analysis of the target group for vaccination (16%), and were somewhat better in persons younger than 65 years (24%) than in the older age group (≥ 65 years; 11%). The point estimates suggested higher VE in outpatients (48%; 95%CI: –1 to 65) than in inpatients (0%; 95%CI: –38 to 27) (Table 2).

**Table 2:** Influenza vaccine effectiveness in preventing laboratory-confirmed influenza A(H3N2) among individuals ≥ 9 years-old in Navarre, Spain, 1 December 2016–31 January 2017 (n = 1,240 patients)

Characteristics	Controls Vaccinated/unvaccinated	Cases Vaccinated/unvaccinated	Crude VE % (95% CI)	Adjusted VE % (95% CI)^a^
**Both healthcare settings**				
All swabbed patients	322/327	240/351	31 (13 to 45)	15 (−11 to 35)
Target group for vaccination^b^	307/218	225/184	13 (−13 to 33)	16 (−12 to 37)
Age group in years				
9−64	56/192	37/239	47 (16 to 66)	24 (−26 to 55)
≥ 65	266/135	203/112	8 (−25 to 33)	11 (−23 to 35)
**Hospitalised patients**				
All swabbed patients	278/205	187/113	−22 (−64 to 9)	0 (−38 to 27)
Target group for vaccination^b^	272/175	185/104	−14 (−55 to 16)	2 (−36 to 29)
Age group in years				
9−64	33/83	14/27	−30 (−179 to 39)	−27 (−188 to 44)
≥ 65	245/122	173/86	0 (−40 to 29)	5 (−34 to 33)
**Primary healthcare patients**				
All swabbed patients	44/122	53/238	38 (3 to 61)	48 (−1 to 65)
Target group for vaccination^b^	35/43	40/80	39 (−10 to 66)	54 (10 to 77)
Age group in years				
9−64	23/109	23/212	49 (4 to 72)	43 (−8 to 70)
≥ 65	21/13	30/26	29 (−70 to 70)	44 (−41 to 78)

CI: confidence interval; VE: vaccine effectiveness.

**^a^** Logistic regression model adjusted for sex, age group (9–24, 25–44, 45–64, 65–85 and ≥ 85 years), major chronic conditions, month of swabbing and healthcare setting (primary healthcare and hospital).

^b^ Target group for vaccination includes people ≥ 60 years old and people with major chronic conditions (body mass index ≥40 kg/m^2^, cancer, liver cirrhosis, dementia, diabetes mellitus, immunodeficiency, heart disease, renal disease, respiratory disease, rheumatic disease and stroke).

In the pooled analysis of all patients, as compared with individuals unvaccinated in the current and four prior seasons, the preventive effect was 61% (95%CI: 30 to 78) in those vaccinated in the current season who had also received 1–2 doses of vaccine in the prior seasons, 24% (95% CI: –6 to 46) in those vaccinated in the current season after 3–4 doses, 42% (95% CI: –5 to 68) in those vaccinated only in the current season, 58% (95%CI: 26 to 78) in individuals without current vaccination but with > 2 prior doses, and 44% (95% CI: 3 to 68) in those unvaccinated in the current season but with 1–2 prior doses. Current and 1–2 dose prior season vaccination, or current season non-vaccination in people with > 2 prior doses showed statistically significant higher protection than current and > 2 prior season vaccinations (Figure). 

**Figure:**
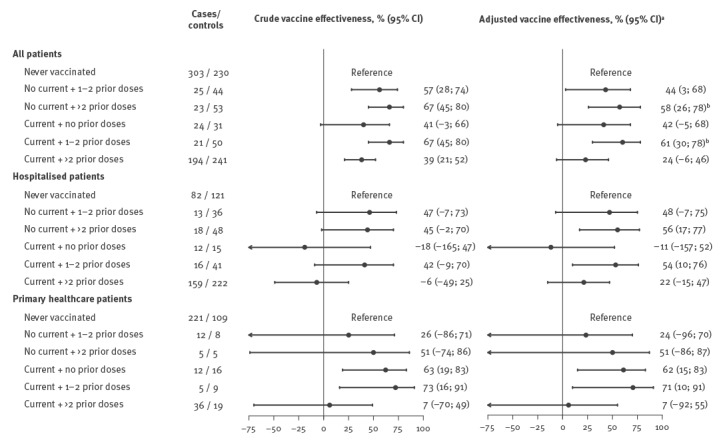
Effectiveness of current season influenza vaccination and of vaccination in the four prior seasons in preventing laboratory confirmed influenza A(H3N2) cases among people ≥ 9 years-old in Navarre, Spain, 1 December 2016–31 January 2017 (n = 1,240 patients)

CI: confidence interval.

^a^ Vaccine effectiveness adjusted by age groups (9–24, 25–44, 45–64, 65–84 and ≥ 85 years), sex, major chronic conditions(body mass index ≥40 kg/m^2^, cancer, liver cirrhosis, dementia, diabetes mellitus, immunodeficiency, heart disease, renal disease, respiratory disease, rheumatic disease and stroke), healthcare setting (primary healthcare and hospital), and month of swabbing.

^b^ p < 0.05 for comparisons with the category ‘Current season vaccination + >2 prior doses’.

In separated analyses of outpatients and inpatients, vaccination only in the current season was protective for primary healthcare consultations but not for hospitalisations. In hospitalised patients however, a history of vaccination in the prior seasons appeared to confer enhanced protection, whether the inpatients were vaccinated or not in the current season (Figure).

## Discussion and conclusion

Estimates of VE during the influenza season help guide health interventions aimed at reducing the impact of influenza in the population [10] and may help in the selection of strains to be contained in the next season’s vaccine. For the 2016/17 season in Navarre, when vaccination status in the prior influenza seasons was not considered, we found low VE (15%) in the whole pool of patients, null VE for hospitalised patients and better protection (48%) for outpatients; the higher protection level in outpatients is consistent with the early estimates reported from the Canadian Sentinel Practitioner Surveillance Network [11]. 

In the analysis considering vaccination history; however, better levels of protection were observed for many of the combinations of current and prior season vaccination, especially for hospitalised patients. The results of the overall analysis suggest that the protective effect of the influenza vaccination against A(H3N2) virus in Navarre in the early 2016/17 season ranged from 24% to 61%, depending on the vaccination status in the current and prior seasons.

The VE estimates were strongly related to the vaccination history. One or two vaccine doses over the four prior seasons maintained or increased the protection of the current season vaccination, but three or more prior doses had a negative interference with the current season vaccine effect. A similar interference was described in previous seasons by other authors [8,12], and inverse exposure-response association has been reported between repeated influenza vaccination and haemagglutinin antibodies titres for A(H3N2) virus [13].

Our results obtained from two independently recruited groups, inpatients and outpatients, were broadly consistent. The main difference was that vaccination only in the current season was protective for influenza cases attended in primary healthcare but not against influenza hospitalisations, which may be due to the poorer immune response among patients that need hospitalisation. Especially remarkable is the preventive effect observed for the vaccine doses received in prior seasons in individuals without current season vaccination.

This study has some limitations. Natural immunity due to exposure to influenza virus was not considered; however, in a previous study we demonstrated that it was not a relevant confounding factor or effect modifier of influenza VE [7]. Since these results are preliminary and have limited statistical power for some analyses, the final results for the season may be different. The study compared laboratory-confirmed cases with controls recruited in the same settings before either patient or physician knew the laboratory result, an approach that reduced selection bias [14]. We included patients recruited in primary care and hospitals, thus achieving representation of the whole spectrum of patients with influenza. The healthcare setting could have acted as a confounding factor, therefore the analyses were adjusted for this variable. This study evaluates a particular situation of circulating virus and composition of the vaccines; caution should be taken in generalising its outcome.

In conclusion, the results suggest that, overall, the different combinations of vaccination in the current and prior seasons were moderately effective against influenza A(H3N2) in the early 2016/17 season in northern Spain. In spite of the possible interferences between the effects of the current season vaccine and frequent prior vaccination, these findings highlight the net benefit of immunisation against influenza.

## Notes

**Primary Health Care Sentinel Network of Navarre:** The members of the Primary Health Care Sentinel Network of Navarre are: I Abad, P Aldaz, R Ansorena, I Arceiz, E Arina, MA Arrechea, I Arribas, MD Artajo, B Azagra, N Aznarez, FC Bartolome, A Beguiristain, A Beltrán, M Bidarte, I Blanco, A Brugos, B Cano, MV Castresana, JC Cenoz, F Cia, B Compains, JR Corpas, F Cortés, B Churío, PC Cuevas, EM Da Costa, MR Díez, FJ Escribano, MJ Esparza, V Etayo, C Fernández Alfaro, B Flamarique, ML Garcés, FJ García Nicolás, AB German, A Giner, JO Guiu, JC Gurbindo, MJ Guruchaga, JA Heras, M Hernández Galindo, MC Hijos, S Indurain, B Iñigo, MC Irigoyen, JJ Jurio, MJ Lizaso, JJ Longás, MJ López, MT Maquirriain, M Mazquiaran, JJ Miner, M Moreno, MA Moros, U Navarro, M Orte, M Oscoz, P Palacio, J Palau, C Pérez Lecumberri, P Pérez Pascual, B Pérez Sanz, A Prado Virto, M Prado Santamaria, A Puig Arrastia M Ramos, BE Rípodas, M Rodríguez, MA Roncal, I Ruiz Puertas, C Sánchez, P Sarrasqueta, F Satrústegui, MA Senosiain, M Sota, ME Ursua, IA Urtasun, M Zardoya, ME Zubieta, F Elia, E Albeniz.

**Network for Influenza Surveillance in Hospitals of Navarre:** The members of the Network for Influenza Surveillance in Hospitals of Navarre are: A Navascués, P Artajo, C Beaumont, X Beristain, J Chamorro, C Ezpeleta, F Gallinas, F Gil, M Herranz, J Hueto, C Martín, L Moreno, S Martínez-Pérez, C Pérez, J Sesma (Complejo Hospitalario de Navarra), MT Ortega, JJ García Irure, M Torres (Hospital Reina Sofía, Tudela), F Lameiro, L Barrado, E Martín (Hospital García Orcoyen, Estella), M Arriazu, M Garcia Cenoz, A Barricarte, J Castilla (Instituto de Salud Pública de Navarra).

## References

[1] European Centre for Disease prevention and Control (ECDC). Risk assessment of seasonal influenza, EU/EEA, 2016-2017 - Update, 25 January 2017. Stockholm: ECDC; 2017. Available from: http://ecdc.europa.eu/en/publications/Publications/Risk-assessment-seasonal-influenza-2016-2017-update.pdf

[2] Sistema de Vigilancia de la Gripe en España. Informe semanal de Vigilancia de la Gripe en España. Semana 5/2017 N° 494. [Weekly report of the influenza surveillance system in Spain 5/2017. No 494]. Madrid: Instituto de Salud Carlos III; 9 February 2017. Spanish. Available from: http://vgripe.isciii.es/gripe/documentos/20162017/boletines/grn052017.pdf

[3] Recommended composition of influenza virus vaccines for use in the 2016–2017 northern hemisphere influenza season. Wkly Epidemiol Rec. 2016;91(10):121-32. 26971356

[4] Recommended composition of influenza virus vaccines for use in the 2015–2016 northern hemisphere influenza season. Wkly Epidemiol Rec. 2015;90(11):97-108. 25771542

[5] Recommended composition of influenza virus vaccines for use in the 2014-2015 northern hemisphere influenza season. Wkly Epidemiol Rec. 2014;89(10):93-104. 24707514

[6] Aguilar I Reyes M Martínez-Baz I Guevara M Albéniz E Belza M Use of the vaccination register to evaluate influenza vaccine coverage in seniors in the 2010/11 influenza season, Navarre, Spain. Euro Surveill. 2012;17(17):20154. 22551499 10.2807/ese.17.17.20154-en

[7] Castilla J Navascués A Fernández-Alonso M Reina G Albéniz E Pozo F Primary Health Care Sentinel Network and Network for Influenza Surveillance in Hospitals of Navarra. Effects of previous episodes of influenza and vaccination in preventing laboratory-confirmed influenza in Navarre, Spain, 2013/14 season. Euro Surveill. 2016;20(22):30243. 10.2807/1560-7917.ES.2016.21.22.30243 27277013

[8] McLean HQ Thompson MG Sundaram ME Meece JK McClure DL Friedrich TC Impact of repeated vaccination on vaccine effectiveness against influenza A(H3N2) and B during 8 seasons. Clin Infect Dis. 2014;59(10):1375-85. 10.1093/cid/ciu680 25270645 PMC4207422

[9] Martínez-Baz I Casado I Navascués A Díaz-González J Aguinaga A Barrado L Effect of repeated vaccination with the same vaccine component against influenza A(H1N1)pdm09. J Infect Dis. 2017; (Forthcoming). 10.1093/infdis/jix055 28453845

[10] Valenciano M Ciancio B I-MOVE study team. I-MOVE: a European network to measure the effectiveness of influenza vaccines. Euro Surveill. 2012;17(39):20281. 23041022 10.2807/ese.17.39.20281-en

[11] Skowronski DM Chambers C Sabaiduc S Dickinson JA Winter A De Serres G Interim estimates of 2016/17 vaccine effectiveness against influenza A(H3N2), Canada, January 2017. Euro Surveill. 2017;22(6):30460. 28205503 10.2807/1560-7917.ES.2017.22.6.30460 PMC5316907

[12] Ohmit SE Petrie JG Malosh RE Fry AM Thompson MG Monto AS. Influenza vaccine effectiveness in households with children during the 2012-2013 season: assessments of prior vaccination and serologic susceptibility. J Infect Dis. 2015;211(10):1519-28. 10.1093/infdis/jiu650 25416812 PMC4462665

[13] Thompson MG Naleway A Fry AM Ball S Spencer SM Reynolds S Effects of repeated annual inactivated influenza vaccination among healthcare personnel on serum hemagglutination inhibition antibody response to A/Perth/16/2009 (H3N2)-like virus during 2010-11. Vaccine. 2016;34(7):981-8. 10.1016/j.vaccine.2015.10.119 26813801 PMC5218812

[14] Valenciano M Kissling E Ciancio BC Moren A. Study designs for timely estimation of influenza vaccine effectiveness using European sentinel practitioner networks. Vaccine. 2010;28(46):7381-8. 10.1016/j.vaccine.2010.09.010 20851086

